# Do All Paths Lead to Mental Fatigue or Is It Merely a Self-Fulfilling Prophecy?

**DOI:** 10.1007/s40279-026-02400-z

**Published:** 2026-03-10

**Authors:** Darias Holgado, Daniel Sanabria, Nicolas Place

**Affiliations:** 1 Faculty of Psychology, UniDistance Suisse, Brig, Switzerland; 2 Institute of Sport Sciences, Quartier, UNIL-Centre, Bâtiment Synathlon, University of Lausanne, Lausanne, Switzerland; 3 Mind Brain and Behavior Research Center, CIMCYC-UGR, University of Granada, Granada, Spain; 4 Department of Experimental Psychology, University of Granada, Granada, Spain

## Abstract

Fatigue, long recognized as a limiting factor in physical performance, has traditionally been conceptualized as comprising both objective (performance fatigability) and subjective (perception of fatigue) dimensions. In sports science, increasing attention has been paid to “mental fatigue”, a state induced by mental efforts, owing to its presumed negative effects on subsequent physical performance. However, this opinion paper argues that the dominant focus on mental fatigue as a singular construct is overly narrow and potentially misleading. Many studies rely on mental tasks (e.g., Stroop test) to induce mental fatigue, assuming that a decline in task performance or self-reported fatigue only equates to mental fatigue. Yet such declines are neither universal nor easily attributable solely to fatigue. Factors such as boredom, mind-wandering, motivation, task automation, and affective states also influence performance and perception during mental tasks. Our research demonstrates that subjective experience during mental tasks is dynamic and multidimensional, with performance decrement and perceived fatigue varying widely across individuals and time. We advocate for a shift from the mental fatigue paradigm to a broader framework, which more accurately captures the interplay between cognitive demands and subjective states. Using tools such as the Temporal Experience Tracing (TET) method, we highlight the complex array of responses during mental tasks that go beyond fatigue alone. In doing so, we challenge current assumptions and call for more nuanced methodologies to assess how mental states affect subsequent physical performance.

## Key Points

**Table:** 

Performing a mental task involves more subjective sensations than mental fatigue.
We argue that this phenomenon warrants re-evaluation within the field of sport sciences and advocate for a more multidimensional approach.
We propose, for instance, framing hypotheses as “The impact of mental task [*X*] on response [*Y*].”

## Introduction

Humans experience what is commonly referred to as “fatigue” when exposed to intense or prolonged exercise [1]. Fatigue is a major topic in the field of sports science because it is considered a limiting factor of physical performance [2]. In sports science, the main focus of attention has mostly been to try to explain the origin of the physical component of fatigue [1, 3]. Fatigue is not straightforward to measure, as it is composed of two dimensions, i.e., one objective (performance fatigability) and one subjective (perception of fatigue) [1, 4, 5]. Fatigability is defined as the rate of change in performance over a given period or against a reference value. The task might be a physical one, for example, a time-to-exhaustion test at 80% of peak power output or a 20 km time-trial on a cycle ergometer, or it might be a mental one, such as a Stroop test [6]. The development of performance fatigability might involve signals at the central nervous system level (for physical and mental activities) and/or impairment beyond the neuromuscular junction (for physical tasks), and the relative contribution of each component depends on the task being performed [7]. In contrast, perception of fatigue would be the result of the central/mental regulation of the activity based on energetic, inflammatory, and neural feedback (homeostatic factors), and the subjective sense of effort related to the cost/value of performing the ongoing task [1, 5]. This cost/value evaluation of a task is independent of the source of sensation of effort but directly linked to its subjective sensation, whether driven by afferent or efferent signals. Tasks perceived as having high value can reduce perceived effort, whereas low-value tasks may feel more effortful even with lower objective demands [8].

Although there is no agreement on what the main determinant is contributing to fatigability and perception of fatigue in response to physical exercise, it is accepted that multiple factors affect this relationship (e.g., metabolic factors [3]). Nonetheless, fatigue is not only induced with physical tasks, but also with mental tasks, which might in turn influence physical performance. Indeed, in recent decades, sports science has become increasingly interested in fatigability, fatigue perception, and how a mental task may impact a subsequent physical exercise [7]. In this context, most studies have used computer-based mental tasks and have assumed that their performance induces a single, distinct state labelled as “mental fatigue.” This state is sometimes treated simultaneously as an isolated sensation and as an umbrella construct encompassing multiple related phenomena [9], which is then presumed to negatively affect subsequent physical performance. Although several researchers acknowledge that additional subjective sensations may contribute to the state of mental fatigue, these sensations are often treated as complementary to mental fatigue rather than as distinct factors with potentially different effects [10]. Furthermore, these sensations are rarely measured within this context, which limits our understanding of their specific roles and potential interactions. In this opinion paper, we argue that this phenomenon requires re-evaluation in the sport sciences domain and advocate for a more multidimensional approach.

## What Has Been Labeled as Mental Fatigue?

Mental fatigue is usually referred to as a complex psychophysiological phenomenon that results in feelings of tiredness or lack of energy following exposure to tasks that require prolonged cognitive exertion [11, 12]. Despite advances in literature in recent years, most of the studies have investigated the effects of performing a mental task on a subsequent physical exercise task by assuming that performing task-taxing executive functions, for example, elicits mental fatigue [13], and would impair subsequent physical performance. Normally, the successful induction of the state of mental fatigue has been assessed by examining changes in mental task performance or variations in subjective experience, i.e., mental fatigue can be quantified in terms of performance fatigability and perceived fatigue in the mental task [1].

At the level of fatigability, mental fatigue has traditionally been quantified objectively by reduced performance of the cognitive task being performed or in a subsequent mental task. For example, the performance in the first half of the task versus the second half of a 30-min Stroop task can be compared [14]. If performance has dropped, it is usually assumed that the task was effective in inducing mental fatigue [14]. Generally speaking, the first thing that comes to mind when someone thinks about mental fatigue is prolonged mental strain over time, but this is not without limitations. Decrements in performance in a mental task depend on various factors such as the person’s goal and motivation for the task, the level of control over the task, the difficulty, and the duration of the task [15, 16]. Even if the tasks are prolonged over time, a decline in performance is not always evident—similar to what occurs with physical exercise [17]. In part, this can be explained by the dynamic nature of performing a cognitive task, and by how cognitive processes are distributed and managed over time [18]. A mental task may become easier and requires less effort as it proceeds, eventually becoming automated, although the effort to remain focused may still be required [16]. The contrary might also be true; according to literature, it takes a greater amount of cognitive processes to maintain the same level of performance as the task progresses [19]. Then, if there is an objective change in mental performance, it is challenging to identify whether the change is due to mental fatigue or caused by other sensations such as effort–reward imbalance [20], lack of motivation [21], sleepiness, or boredom [22].

This ambiguity arises because similar tasks are often used to assess constructs that are defined by overlapping outcomes, particularly reductions in performance. For instance, cost/value models explain decreases in performance, dropout, or withdrawal as shifts toward behaviors perceived as more valuable or rewarding, rather than as direct manifestations of fatigue [23]. In boredom research, rising boredom is associated with reduced effort investment and increased reports of difficulty, frustration, and tiredness; and higher boredom also predicts poorer performance on secondary tasks [24]. Similarly, the Integrative Model of Effortful Control proposes that effortful control entails intrinsic costs linked to a gradual decline in the functional connectivity of neural networks supporting sustained effort, from which mental fatigue is thought to emerge [25]. Although these models and perspectives approach the issue from different angles, they converge on the observation of cognitive performance decline as a common outcome. Each emphasizes one predominant feeling or mechanism, yet multiple sensations may contribute simultaneously, depending on individual and contextual factors. This tendency may partly reflect researchers’ focus on isolated constructs without fully considering the range of subjective experiences elicited by the tasks themselves. In other words, for example, what comes first, the mental fatigue that triggers the boredom or the boredom that triggers the mental fatigue?

Additionally, more objectively, it has been shown that participants performing high-demand cognitive tasks throughout the day had higher concentrations of glutamate, which in turn might contribute to the onset of mental fatigue [23]. Moreover, increase in neurotransmitter concentration, such as adenosine, has been associated with mental fatigue, although the evidence is scarce [26]. Neurophysiological measures such as electroencephalography (EEG) or pupillometry might also provide valuable complementary information to assess subjective states. However, these measures do not directly represent any specific subjective experience, as their interpretation ultimately depends on the particular questions asked of participants [27]. Much of the existing literature makes direct inferences by assuming that, if participants report a certain state, the corresponding physiological change must reflect that state [28].

In any case, in the studies considering the role of mental fatigue in sports performance, participants are classically asked to rate their level of mental fatigue before the cognitive task and prior to the physical effort to ensure that the task induced mental fatigue. If the reported mental fatigue is higher in the experimental condition than in the control condition, the manipulation is deemed to be successful. However, what if, instead of asking about mental fatigue, participants would have been asked about a subjective state of boredom or whether they were just fed up with the task [27]? Together with mental fatigue, or even without any change in mental fatigue, it is likely that cognitive tasks induce other unmeasured or unreported subjective experiences, underscoring the complexity of studying mental fatigue beyond task difficulty or performance alone [27]. A limitation of self-reported measures is that individuals have limited insight, social desirability bias, and a variety of mappings to gauge their cognitive state. Moreover, it is important to identify the factors and causes that people identify as mentally draining [29]. For example, in an elite scenario, some athletes complain about press conferences, sponsorship, etc., as potential factors impacting their mental state [30]. This does not necessarily mean that these factors or any other mental tasks such as the ones in those laboratory studies are mentally fatiguing, but participants are typically only asked about mental fatigue.

## Do All Paths Lead to Mental Fatigue?

Self-fulfilling prophecies are predictions that come true partly because they are believed to be true [31]. A second assumption underpinning the argument for self-fulfilling prophecies is that even if the effects are small, they may accumulate over time, making the overall effect quite large [13, 31]. While it is plausible that mental fatigue can have a negative effect on a subsequent physical performance, is it only mental fatigue that is experienced when performing a cognitive task [29, 32]? When researchers focus on a single psychological dimension, that variable is likely to emerge as a predictor largely because alternative explanations are excluded. In contrast, what happens when one asks about other psychological dimensions associated with the performance of a mental task on a computer? This is of special relevance, as the Stroop task has been used in different studies to examine either boredom, mind-wandering, or mental fatigue, for example [24, 33, 34].

**Fig. 1:**
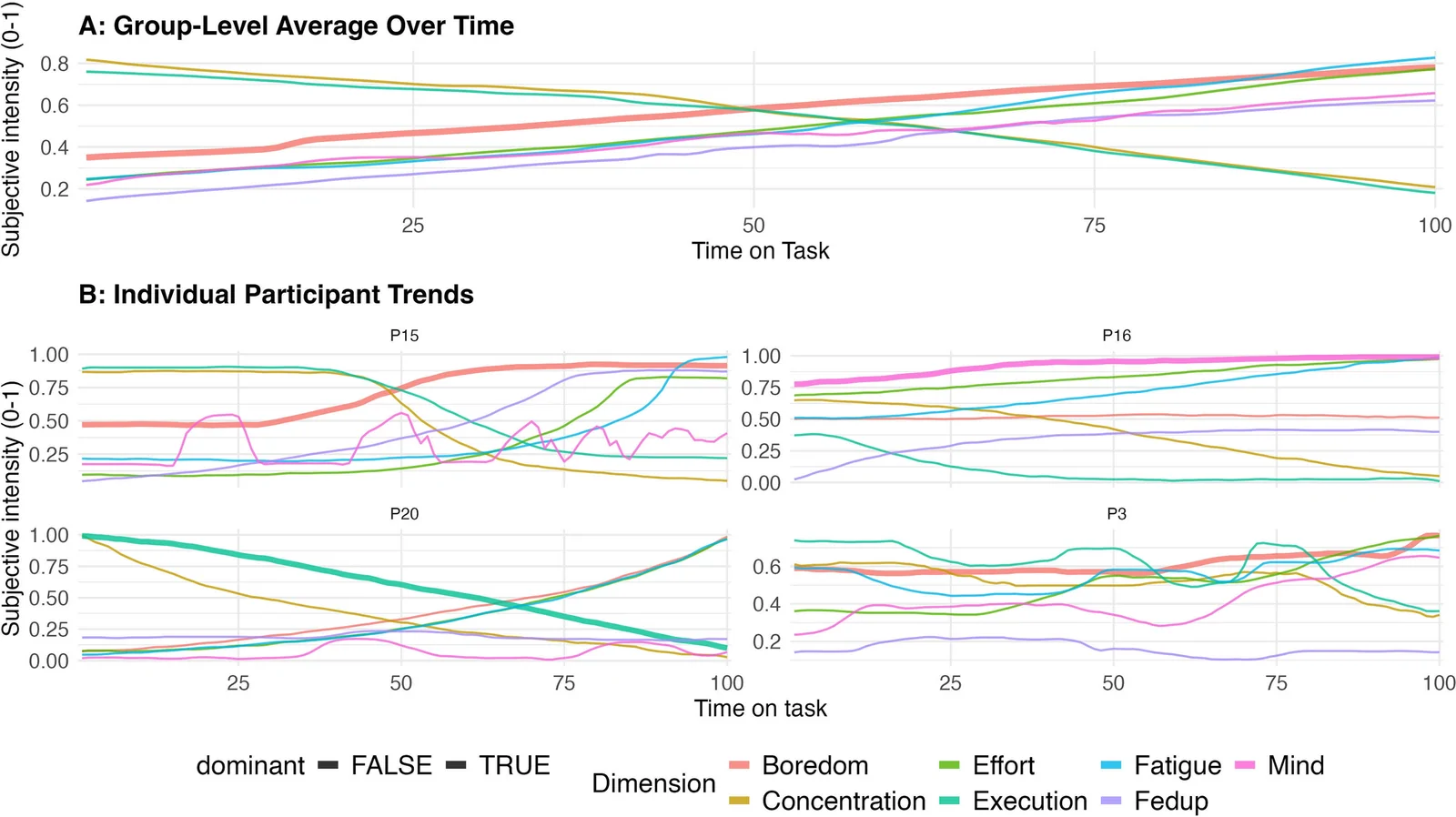
(**A**) Group average trend of each subjective dimension during the execution of a TloadDback task until cognitive failure (*x*-axes represent the relative total time). Each line represents a subjective dimension, and the dimension with more weight across the task (boredom) is highlighted. The first part of the task was characterized by low levels of mental fatigue, boredom, mind-wandering, effort, and frustration, but as the task progressed, the opposite state was more prevalent. (**B**) Representation of participants randomly chosen from the group. Data from Holgado et al., 2026 [35]

In a series of studies, we have shown that the performance of a cognitive task is a dynamic and multidimensional experience and cannot be attributed to the aspect of mental fatigue alone [27, 35]. Even if the tasks are prolonged over time, performance decrement is not always evident. To solve this limitation and better understand the subjective experience, we established an objective criterion of fatigability in the mental task, i.e., participants had to complete the task until performance dropped below a predefined threshold (without the participants being aware of the objective criterion). This method ensures that participants reach their cognitive limit by objectively tracking performance fatigability through a moving average window, rather than evaluating progress block by block. This provides a dynamic, individualized measure of cognitive load. In doing so, we also limit the gradient effect or end spurt, i.e., participants spend more effort when the task is about to finish (as occurs with physical exercises) [36, 37]. We evidenced that time to mental failure varied across participants (e.g., ranging from 9 to 120 min), and independently of mental task duration, the subjective experience was dynamic across the task [27, 35]. Likewise, we employed a self-selected documentary as the control condition, aiming to minimize the potential influence of boredom or mental fatigue that might arise from other cognitive activities. This approach appeared effective in maintaining a relatively neutral state for most of the control period [27]. We have introduced the Temporal Experience Tracing (TET) method in this field, allowing us to consider a much broader variety of subjective experiences across time and without prior assumptions about their impact on task performance [38, 39] (Fig. 1). The combination of a cognitive task individualized in terms of cognitive load (both duration and intensity) and performed until failure, together with the detailed subjective data obtained through the TET, provided a more nuanced understanding of this topic. This approach does not assume that a single psychological dimension is primarily responsible for the potential negative effects of subjective states on subsequent exercise.

Our studies [27, 35] highlighted the complex nature of the subjective experience linked to cognitive tasking, while failing to report evidence of the purported effect of performing a cognitive task prior to a physical task. They add to the mixed findings in literature. Indeed, there are studies reporting that engaging in a mental task prior to physical exertion may impair subsequent performance [14, 40], and there are also studies that have failed to replicate this effect [17, 41, 42]. In any case, in this opinion piece, we do not aim to resolve the debate regarding the presence or absence of such an effect (for further discussion, see [13]), but instead argue that the current terminology and methodological approaches may be inadequate. Note also that the present work does not attempt to provide a precise definition of mental fatigue, as the construct is inherently complex. Instead, mental fatigue is conceptualized as a multidimensional phenomenon that manifests across subjective experience, behavioral performance, and physiological processes. Mental tasks elicit a range of subjective experiences beyond mere mental fatigue, just as physical tasks involve more than the perception of effort, such as the affective responses to exercise-induced pleasure [43]. Rather than referring broadly to mental fatigue, we advocate first for understanding which subjective sensations are elicited for the classical task used in those studies, as it encompasses a wider array of cognitive and affective responses beyond fatigue alone. It is essential that researchers ensure the task in question indeed imposes a measurable mental/cognitive load, and that they assess the relevant subjective feelings in that particular context. Otherwise, we propose for instance framing hypotheses as “The impact of mental task [*X*] on response [*Y*]” (for example, “The impact of Strop task on a 30 km cycling time-trial”). This is important because any observed effects on physical performance may not be attributable solely to mental fatigue but rather to a complex interplay of cognitive and emotional factors.

## Conclusions

Although not neglecting that mental fatigue can have an impact on performance, task execution may elicit a wide range of cognitive/mental/psychological changes, and a single dimension, such as mental fatigue, is unlikely to fully explain the potential impact of performing a cognitive task on a computer on a subsequent physical exercise. The prevailing understanding of mental fatigue warrants reconsideration, as the present findings [27, 35, 44], together with those of previous research [45–47], suggest that mental tasks encompass a broader range of experiences beyond mental fatigue alone. Previous studies have predominantly reported increased feelings of mental fatigue, likely owing to the limited scope of measurement focused solely on that sensation. Then, it is important to reconsider the subjective sensations elicited by typical laboratory tasks, as these are often assumed rather than empirically verified. Whereas the cognitive functions involved in those tasks are well established [48, 49], in most studies, researchers presuppose that a given task evokes a specific feeling. However, participants are rarely asked directly about the sensations that they actually experience. In other words, they are asked to tell us what we want to hear (self-fulfilling prophecy). This highlights a potential methodological bias and emphasizes the importance of adopting a more comprehensive, multidimensional framework for examining subjective cognitive states.
